# Study design and baseline results of an open-label cluster randomized community-intervention trial to assess the effectiveness of a modified mass deworming program in reducing hookworm infection in a tribal population in southern India

**DOI:** 10.1016/j.conctc.2016.12.002

**Published:** 2016-12-10

**Authors:** Rajiv Sarkar, Anuradha Rose, Venkata R. Mohan, Sitara S.R. Ajjampur, Vasanthakumar Veluswamy, Rajan Srinivasan, Jayaprakash Muliyil, Vedantam Rajshekhar, Kuryan George, Vinohar Balraj, Nicholas C. Grassly, Roy M. Anderson, Simon J. Brooker, Gagandeep Kang

**Affiliations:** a Division of Gastrointestinal Sciences, Christian Medical College, Vellore, 632004, Tamil Nadu, India; b Community Health Department, Christian Medical College, Vellore, 632002, Tamil Nadu, India; c Department of Neurological Sciences, Christian Medical College, Vellore, 632004, Tamil Nadu, India; d Society for Applied Studies, No. 14, Natteri Krishnamachari Street, Krishna Nagar, Vellore, 632001, Tamil Nadu, India; e Department of Infectious Disease Epidemiology, School of Public Health, Faculty of Medicine, Imperial College London, Norfolk Place, London, W2 1NY, United Kingdom; f Faculty of Infectious and Tropical Diseases, London School of Hygiene and Tropical Medicine, Keppel Street, London, WC1E 7HT, United Kingdom

**Keywords:** Hookworm, Mass deworming, Cluster randomized trial, Mathematical modeling, India

## Abstract

**Introduction:**

Hookworm infection is a leading cause of iron deficiency anemia and malnutrition in resource-poor settings. Periodic mass deworming with anthelminthic drugs remains the cornerstone of hookworm control efforts worldwide. Reinfection following treatment occurs, reflecting the human host's inability to acquire immunity following exposure to an untreated reservoir of infection. This cluster randomized trial will evaluate the effectiveness of a modified, population-based, mass deworming strategy in reducing hookworm infection in an endemic southern Indian population.

**Methods:**

Forty five tribal villages were randomized into three groups: one received annual treatment; the second received two rounds of treatment at 1-month intervals; and the third received four rounds of treatment – two rounds 1 month apart at the beginning, followed by another two after 6 months. Stool samples collected through cross-sectional parasitological surveys pre- and post-intervention, and at 3-monthly intervals for a period of 1 year were tested for presence of hookworm ova. Long-term effectiveness of treatment will be assessed through another survey conducted 2 years after the last treatment cycle.

**Results:**

From a population of 11,857 individuals, 8681 (73.2%) were found to be eligible and consented to participate, out-migration being the primary reason for non-participation. Baseline stool samples were obtained from 2082 participants, with 18.5% having hookworm infection, although majority were low intensity infections (<2000 eggs per gram of feces).

**Discussion:**

This study will help identify the optimal mass deworming strategy that can achieve the greatest impact in the shortest period of time, particularly in settings where long-term program sustainability is a challenge.

## 1 Introduction

Hookworms, along with the other soil transmitted helminths (STH), *Ascaris lumbricoides* and *Trichuris trichuira*, are among the commonest gastrointestinal infections in humans [1]. Hookworms affect an estimated 438.9 million people worldwide, resulting in 3.2 million disability adjusted life years (DALY) [2]. This exceeds the disability burden of most other tropical diseases [3], [4]. Most human hookworm infections are caused by two species - *Necator americanus* and *Ancylostoma duodenale*, although considerable regional variation in the species composition has been described [5]. There is a clear relationship between hookworm prevalence and low socioeconomic status, with the majority of “at risk” population living on less than US$2 per day [6], [7].

In children, hookworm infections are associated with iron-deficiency anemia, stunted growth, poor nutritional status and reduced physical and cognitive abilities, and therefore may profoundly impact school performance and future economic productivity [8], [9]. Increasing hookworm infection intensity is also associated with lower hemoglobin levels in adults, particularly in pregnant women living in low-income countries [10], [11].

The World Health Organization (WHO) has set a global target of regularly treating at least 75% of the pre-school and school-aged children in endemic areas through school-based deworming programs to eliminate morbidity due to STH by 2020 [12]. Despite high cure rates [13], [14], failure to prevent reinfection even after effective treatment is a recognized shortcoming of this strategy [15], [16], [17], [18], often due to an untreated reservoir of infective stages in the environment. In a meta-analysis, a 30% reinfection rate was observed at 3 months post-treatment, increasing to 57% at 12 months [19]. A positive correlation between pre-treatment infection intensity and reinfection status has also been noted [18]. Moreover, recent modeling-based estimates suggest that school-based deworming may have limited impact in interrupting the community transmission of STH infections, especially in places where hookworm predominates because most infection is harbored by adults [20]. The transmission of an infectious agent following drug treatment is a dynamic process, and is determined by many factors including treatment frequency, coverage and efficacy [21], [22]. Suboptimal treatment may result in persistence of an untreated reservoir of transmission, thereby increasing the likelihood of reinfection [23] and the need for periodic treatments to interrupt transmission in endemic communities [24]. This, in turn, raises the question of long-term sustainability of such programs and the possibility of emergence and spread of drug resistance [25], [26]. There is a need for studies comparing different regimens of mass drug administration (MDA) against hookworm infection to identify optimal deworming strategies that can minimize morbidity or interrupt transmission, thereby enabling the MDA to be stopped.

The primary objective of this study is to compare the hookworm reinfection rates between a population-based MDA strategy of an annual treatment cycle (with albendazole) with two and four cycles of treatment respectively, for a period of 1 year in an endemic tribal population in southern India [27]. The secondary objective is to identify individual, household, community and spatial correlates of infection; and to develop predictive models to understand the dynamics of hookworm transmission in endemic populations. The highly aggregated nature of worm distribution within human communities and predisposition to reinfection among those heavily infected is well established [20], [28].

## 2 Materials and methods

### 2.1 Study setting

**Fig. 1:**
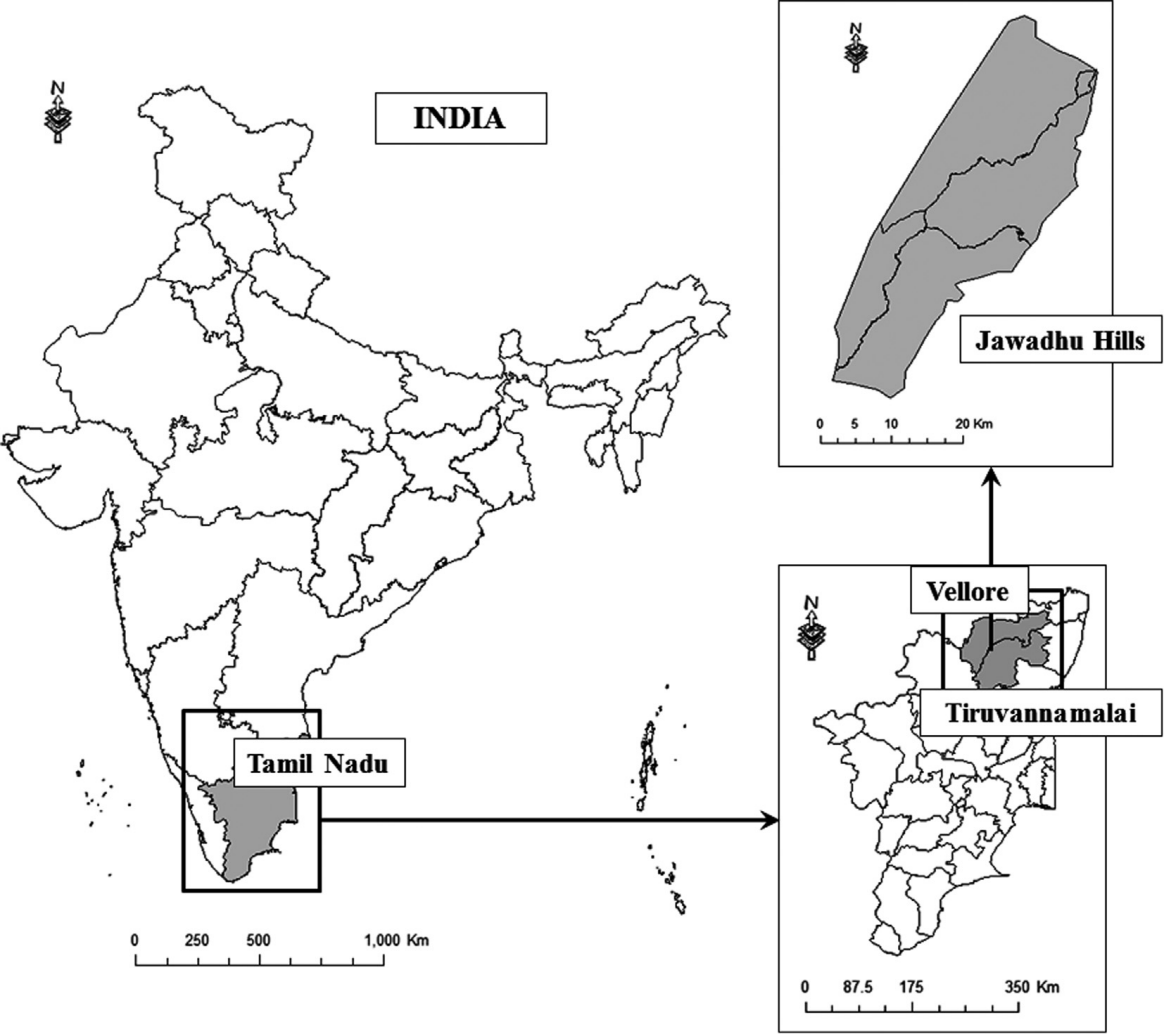
Map showing location of the study area.

This ongoing study is being conducted in the Jawadhu Hills (JH) block, at the borders of Vellore and Tiruvannamalai districts of Tamil Nadu in southern India (Fig. 1), with a population of about 80,000, mostly tribal with a common ancestry. It has 11 panchayats with about 250 villages; each village has between 15 and 100 households. The community is predominantly agrarian, cultivating rice, millet and maize; pig rearing is a common practice. During non-agricultural seasons, many people migrate to coffee plantations in the neighboring states as short-term (temporary) laborers.

The area has a hilly terrain with poor road access, lack of safe drinking water and poor sanitation facilities. It is endemic for lymphatic filariasis, and all residents aged >1 year are under a mass treatment program with annual co-administration of diethylcarbamazine (DEC) and albendazole since 2007, as part of the Government of India (GoI) initiative to eliminate lymphatic filariasis [29]. Despite this, a cluster survey of 1237 subjects from 680 households in 2011 found a very high hookworm burden, with an overall prevalence of 38%. As is typical for hookworm infection, the prevalence and intensity of infection increased with age [27].

### 2.2 Study design

**Fig. 2:**
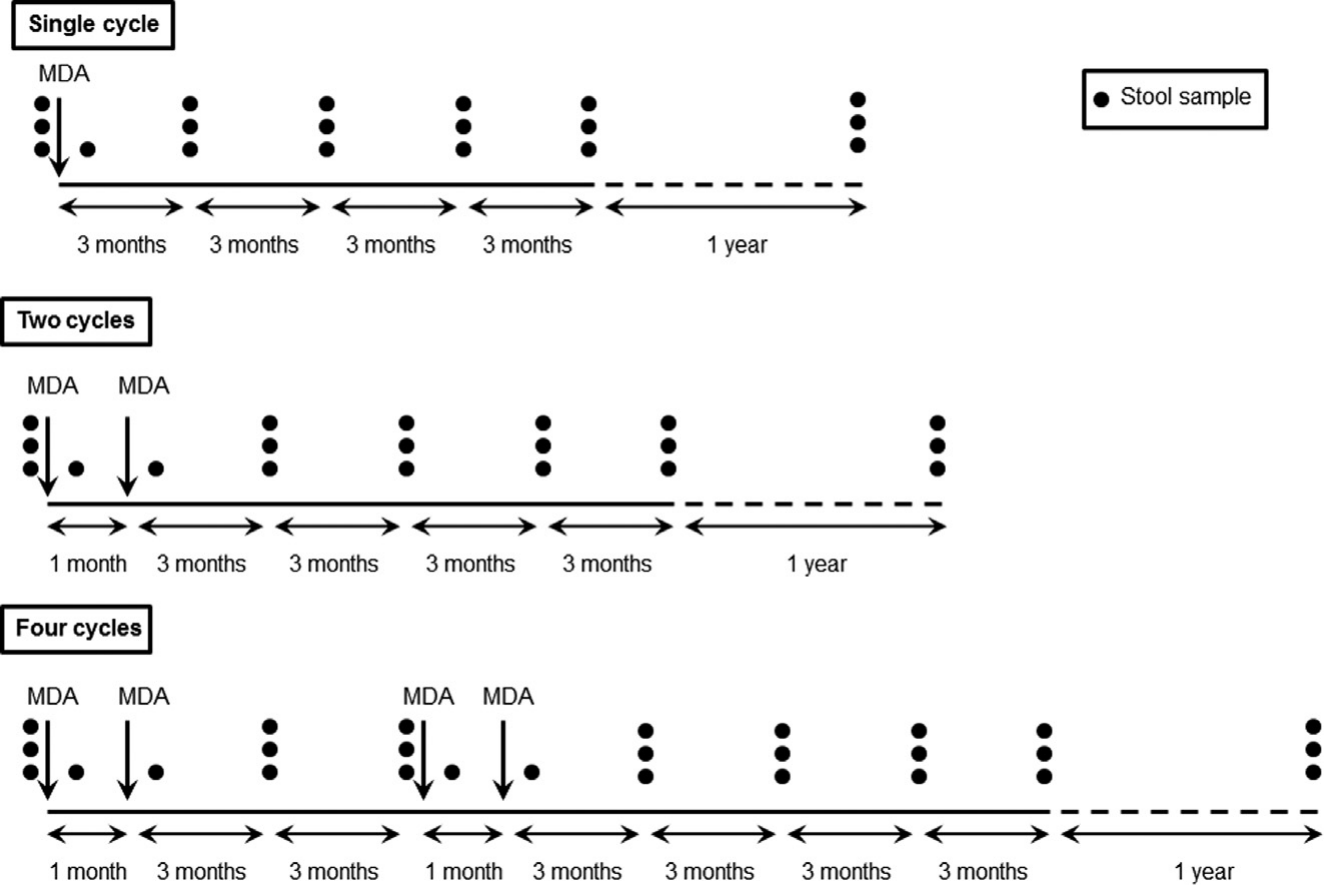
Schematic diagram representing the timelines for mass drug administration (MDA) and stool sample collection in the three intervention groups. The black dots represent the number of stool samples collected per participant at each time point. The stool samples are collected through multiple cross-sectional parasitological surveys. The dashed line represents the time elapsed between the primary data collection and the follow-up survey.

The study is an open-label, cluster randomized, community-intervention trial. A total of 45 villages (clusters) with similar population structure, water supply and sanitation practices were selected. The villages were randomized into one of three groups (Fig. 2):

1. Single cycle (15 villages): Village residents of all ages in this group received only one cycle of 400 mg albendazole, at month 1.
2. Two cycles (15 villages): Village residents of all ages in this group received two cycles of 400 mg albendazole at 1 month interval, at month 1 and 2.
3. Four cycles (15 villages): Village residents of all ages in this group received two cycles of 400 mg albendazole at month 1 and 2, followed by another two cycles at month 8 and 9 (a total of four cycles).

The timing of the MDA cycles in the 2- and 4-cycle groups is based on the hookworm biology. The released rhabditiform larvae take about 5–10 days in the soil to become the infective filariform (third-stage) larvae [30]. Hence, it was hypothesized that a second MDA cycle (in the 2-cycle group) covering the extrinsic incubation period will reduce reinfection from the infective larvae already present in the soil. However, as the larvae are known to survive in soil for weeks under favorable environmental conditions [31], it was speculated that these two treatment cycles may not be adequate to achieve transmission breakpoint in endemic settings; therefore another intervention group (the 4-cycle group) with two additional MDA cycles after 6 months was added (Fig. 2). Moreover, multiple treatment cycles will result in an overall increase in MDA coverage and a subsequent reduction in worm intensity, thereby reducing the need for repeated treatments.

### 2.3 Interventions

All study groups received albendazole 400 mg, an anthelminthic drug that is highly efficacious against hookworm infection [13]. The only difference between the three study groups is the frequency of treatment, which is outlined in Fig. 2. In order to mimic the population-based MDA campaigns initiated by the GoI for elimination of lymphatic filariasis [29], designated deworming days were planned for each village and the residents informed well in advance. Everyone present in the village, who were otherwise eligible and provided written informed consent, were administered a single dose of albendazole 400 mg through door-to-door household visits. A mop-up campaign was conducted the subsequent day to ensure maximum coverage. Participants were interviewed the subsequent day to record details of tablet consumption, and followed up repeatedly for a period of 1 week post-treatment to record any adverse/serious adverse events following intervention.

### 2.4 Outcome measures

The effectiveness of treatment was evaluated qualitatively, based on the prevalence of hookworm infection, and quantitatively based on the intensity of infection (fecal egg count). Prevalence and intensity of infection at the end of 12 months after the last MDA cycle is the primary outcome measure for this study. Secondary outcome measures include prevalence and intensity of infection after each treatment cycle and at 3, 6, and 9 months post-treatment. Long-term effectiveness of the treatment on prevalence and intensity of infection will be assessed through a follow-up cross-sectional parasitological survey 2 years after the last MDA cycle.

### 2.5 Sample size

The sample size was calculated based on following assumptions: 38% prevalence of hookworm infection at baseline [27]; CR of 75% [14]; reinfection rate of 60% at the end of 1 year [19] in the single cycle group; a minimum effect size of 30%; power of 80%; alpha error of 5%; and 10% refusal rate. This requires approximately 165 participants in each group [32]. Since this is a cluster randomized trial, the sample size was inflated to account for the increased variance due to clustering of hookworm cases at village and household levels [33]. Analysis of data from two previous cross-sectional studies in Tamil Nadu [27], [34] showed an intra-cluster correlation coefficient of 0.06 for hookworm infection, which translated into a design effect of 2.7 for a cluster-size of 30. Based on this, a total sample size of 450 participants per intervention group (single/two/four cycles) was considered adequate to detect effectiveness of the intervention regimes, both qualitatively and quantitatively. Thus, at least 1350 participants in 45 clusters (30 in each cluster) had to be contacted during each parasitological survey (Fig. 2).

### 2.6 Identification of villages and participant recruitment

The villages covered by the Community Health Department of the Christian Medical College (CMC), Vellore, have been mapped, and the geodatabase includes coordinates of each house along with basic socio-demographic data. Additional village-level spatial attributes include the streets, water distribution system, latrines and outdoor defecation areas. This geodatabase was used to identify villages with similar WASH (water, sanitation and hygiene) facilities, proportion of households engaged in agriculture-related activities and proportion of households with domesticated animal(s) for inclusion in the study. Very large villages (population ≥1000), non-rural settlements or villages with non-tribal population were excluded.

The study was initiated after discussions with village leaders and the local administration. Once permission was obtained, a short health educational module about STHs, their transmission and prevention was conducted in each village. A brief description of the study was also provided at the same meeting. Thereafter, the villagers were approached individually for participation and written consents obtained from all participants or their parents/legal guardians.

A person was eligible to participate if he/she was a permanent resident of the village, was aged between 2 and 70 years (both years inclusive), and was willing to provide informed consent and have study personnel visit at home. Individuals with syndromic or serological evidence of HIV infection or immuno-compromise, pregnant women, those with a history of seizure, epilepsy or known neurological disorder, and those with known history of hypersensitivity to albendazole were excluded from participation.

### 2.7 Baseline data collection and follow-up

Detailed information on socio-demographic and hygiene characteristics were collected from all participants at baseline through a door-to-door survey of the households. Stool samples from a subset of participants were collected at baseline, after each round of MDA, and at 3-monthly intervals until the end of 1 year. A follow-up survey will be conducted at the end of 2 years after the last MDA cycle to assess long-term effectiveness of the treatment. The stool sample collection schedule (including the planned follow-up parasitological survey) is presented in Fig. 2. Because of the seasonal migration pattern of the study population, the parasitological surveys (pre-MDA/post-MDA/follow-up) were conducted as separate cross-sectional studies.

### 2.8 Laboratory methods

All stool samples were examined for the presence of helminth ova by microscopy using saline and iodine wet preparations before and after concentration by the formol-ether technique [35], which has a sensitivity of 80% for parasite detection using three samples [36]. Those found positive for hookworm were re-examined by the McMaster egg counting technique to quantify the number of eggs/g of stool (fecal egg count) [37]. The species of hookworm isolates were determined by PCR-RFLP based methods [38].

### 2.9 Data management

Structured questionnaires (case report forms, CRFs) were used to collect field data. All CRFs were piloted in the community. Data collection was standardized through extensive training of the field staff prior to commencement of the study and periodically during the follow-up. Data were entered in duplicate and missing values or discrepancies between the two data entry sets send back to the field site for resolution. Identical values were saved to a master database in a central server, with a network backup system.

### 2.10 Statistical analysis

All variables will be examined using descriptive statistics (measures of central tendency [means and medians], dispersion [standard deviations, interquartile ranges]) for continuous variables, frequency counts and marginal percentages [with 95% confidence intervals] for categorical variables). The success of randomization will be evaluated by comparing the baseline characteristics between the treatment and control groups; no formal statistical testing will be performed as per the CONSORT (Consolidated Standards of Reporting Trials) recommendation [39]. The primary analysis will be an intention-to-treat analysis comparing prevalence and intensity of infection between the three groups at the end of 12 months after the last MDA cycle, adjusted for age and gender. Generalized estimating equations (GEE) will be used to account for the correlated data structure [40]. The prognostic effect of variable(s) with noticeable baseline imbalances will be assessed by comparing results of analysis with and without adjustment for such variable(s) [41]. The effect of clustering at village and household levels will be explored through multilevel (hierarchical) modeling [42]. In all analyses, past research demonstrates much variability in fecal egg counts (negative binomial distribution); this will be accounted for where appropriate.

### 2.11 Mathematical model building

Mathematical models have extensively been used to understand the dynamics of helminth transmission and to investigate the effectiveness of MDA in controlling infection [43], [44], [45]. When backed by robust field data, they have great potential to enhance the design, and hence effectiveness, of disease and infection control programs.

The data collected from this study (pre- and post-treatment fecal egg count, prevalence of hookworm infection, and per capita reinfection rates in the age and gender groups within the treatment groups) will provide the key parameters to build the hookworm transmission models. The effect of different MDA regimes on hookworm transmission will first be assessed using established deterministic age-structured models [46] and individual-based stochastic models [47]. The complex host-parasite interaction on transmission and drug efficacy at the individual, household and community level will then be explored using simulation techniques based on the stochastic model. Particular attention will be given to the impact of migration for temporary employment, and individual compliance over multiple treatment events.

### 2.12 Ethical considerations

This study has been approved by the Institutional Review Board (IRB) of CMC, Vellore, India and is registered in the Clinical Trials Registry - India (CTRI: http://ctri.nic.in) under registration number CTRI/2013/05/003676. Written informed consent was obtained from all participants prior to participation. For children <18 years of age written informed consent was obtained from their parents/legal guardians; additionally, assent was obtained from all children aged 8–17 years.

## 3 Results

The total population was 11,857, with a sex ratio of 960 females per 1000 males. The median (IQR) population per village was 238 (203–289). The socio-demographic profile of the households in the study villages (collected through the baseline survey) is presented in Table 1. There were a median (IQR) of 4 (3–5) members per household, with 92.2% households having one or more members engaged in agriculture-related activities. Majority (81.9%) of the families sourced their drinking water from public supplies (tap or borewell); only 0.3% had a functional toilet in the house.

**Table 1:** Socio-demographic characteristics of the study villages (N = 45) obtained through the baseline survey.

Demographic characteristic	Number
Total number of households	2770
Number of females per 1000 males	960
Median (IQR) family size	4 (3–5)
Household socio-economic status	
Low	764 (27.6%)
Middle	1326 (47.9%)
High	680 (24.6%)
Number of households with at least one member engaged in agriculture-related activities	2555 (92.2%)
Number of households with a functional toilet	7 (0.3%)
Public tap or borewell as the primary source of household drinking water	2269 (81.9%)
Number of households reporting having one or more domesticated animal or having members coming in prolonged contact with animals	2292 (82.7%)

Recruitment of participants commenced in October 2013 and was completed in November 2014. Of the total population, 8681 (73.2%) were found to be eligible and provided informed consent. Primary reason for non-participation was male migration out of the study area for temporary employment: 1947/11,857 (16.4%) residents were not present in their respective villages during the entire study period. Only 1.3% of the residents (149/11,857) declined to participate (Table 2).

**Table 2:** Details of enrollment in the study villages (N = 45).

	Number
Total population	11,857
Excluded participants	3176
Migration (out of area)	1947
Did not fulfil inclusion/exclusion criteria	1080
Declined to participate	149
Enrolled participants	8681

Baseline stool samples were obtained from a total of 2082 participants, with a median (IQR) number of 46 (44–48) participants per village providing one or more stool samples; 1727 (82.9%) provided all 3 stool samples as per protocol. Three hundred and eighty six (18.5%) of the 2082 participants were positive for hookworm with a mean (SD) intensity of 678 (1649) eggs per gram (epg) of feces. The majority (361, 93.5%) had low intensity infection (<2000 epg) and only 12 (3.1%) hookworm positive individuals excreted ≥4000 epg (high intensity infection).

The socio-demographic profile of enrolled participants from whom baseline stool sample(s) was obtained and those from whom baseline stool sample was not obtained is outlined in Table 3. Comparison of their socio-demographic profile revealed some differences. Participants providing stool samples tended to be younger (*P* < 0.001), were less likely to engage in agriculture-related activities (*P* < 0.001) and attained slightly lower educational levels (*P* = 0.069). They were, however, comparable in terms of gender distribution (*P* = 0.135), socio-economic status (*P* = 0.150), usage of public taps (*P* = 0.693) and toilet usage (*P* = 0.917).

**Table 3:** Comparison of socio-demographic characteristics of enrolled participants from whom baseline stool sample was obtained (n = 2082) with those from whom baseline stool sample was not obtained (n = 6599).

	Baseline stool sample		*P*-value^b^
	Obtained	Not obtained	
Age (in completed years)^a^			
<5 year	131 (6.3)	243 (3.7)	<0.001
5–14 year	642 (30.8)	1418 (21.5)	
15–44 year	997 (47.9)	3893 (59.0)	
≥45 year	312 (15.0)	1045 (15.8)	
Gender^a^			
Male	1021 (49.0)	3360 (50.9)	0.135
Female	1061 (51.0)	3239 (49.1)	
Socio-economic status^a^			
Low	423 (20.3)	1393 (21.1)	0.905
Middle	1089 (52.3)	3344 (50.7)	
High	570 (27.4)	1862 (28.2)	
Engaged in agriculture-related activity^a^			
Yes	1120 (53.8)	4003 (60.7)	<0.001
No	962 (46.2)	2596 (39.3)	
Level of education^a^			
No formal schooling	956 (45.9)	3074 (46.6)	0.069
Primary school (1–5 years)	583 (28.0)	1388 (21.0)	
Middle school (6–8 years)	311 (14.9)	1012 (15.3)	
High school (9–10 years)	136 (6.5)	671 (10.2)	
Higher secondary (11–12 years)	62 (3.0)	285 (4.3)	
College & above (>12 years)	34 (1.6)	169 (2.6)	
Functional toilet in the house^a^			
Present	6 (0.3)	20 (0.3)	0.917
Absent	2076 (99.7)	6579 (99.7)	
Public tap as source of household drinking water^a^			
Yes	1852 (89.0)	5343 (81.0)	0.693
No	230 (11.0)	1256 (19.0)	

a Numbers in parenthesis represents column percentage.

b Adjusted for clustering at village and household levels.

## 4 Discussion

It is estimated that approximately 71 million Indians are infected with hookworms, accounting for 12% of the global disease burden [48]. Per the 2001 census, 8.2% of the Indian population is tribal [49], mostly living in remote locations with poor transport/road access and low health-service coverage. Poverty and lack of sanitation facilities make them vulnerable to high levels of hookworm infection. There is therefore an urgent need to explore effective STH control strategies in this and similar settings within India.

The importance of safe water, adequate sanitation and hygiene in preventing transmission of STH infection is well documented [50], [51]. However, there are several barriers to successful implementation of WASH interventions in communities with poor sanitation coverage [26]. In rural Indian communities, cultural and behavioral aspects such as habits, socializing patterns, social customs and daily routines, rather than absence of toilet infrastructure, plays an important role in the community preference for open defecation [52], [53]. Given these impediments to the rapid implementation and uptake of WASH interventions in STH-endemic communities, periodic MDA with anthelminthic drugs remains the “first-line rapid control measure” for STH infections [24].

This community-intervention trial is being conducted in a “difficult-to-reach” tribal population in southern India. Despite several operational challenges such as a difficult hilly terrain, high rates of seasonal migration and cultural practice of not letting “outsiders” (non-residents) during important community activities (village festivals, marriages or deaths), a high recruitment rate could be attained - 73.2% of the eligible population consented to participate. Minor differences between the socio-demographic profile of participants providing and not providing the baseline stool sample(s) reflects a potential non-response bias. This was, however, inevitable, given the study design and the migration pattern of the study population (seasonal, short-term migration to neighboring states as plantation workers). The biggest challenge to the design of an effective MDA-based hookworm control strategy lies in migration due to employment, which may have a great impact on the effective MDA coverage. Detailed data on these migration patterns will help devise strategies to treat the migrant population.

The lower than expected baseline prevalence of hookworm infection is a potential limitation of this study and can increase the chance of type I (α) or type II (β) error due to the reduced statistical power to detect the hypothesized effect size [54]. This was unanticipated as the expected prevalence for the sample size calculation was based on an earlier study conducted in the same geographical area (involving a different set of villages) [27]. Given the common ancestry of the population, and similarities in WASH practices, occupation and lifestyle characteristics, this difference in the prevalence of hookworm infection between the present and the earlier study underscores the importance of peridomestic environment in hookworm transmission, which should be investigated further.

The goal of study is to integrate empirical field data with statistical and mathematical modeling to identify the optimal hookworm control strategy that can achieve the greatest impact in the shortest period of time, particularly in settings where long-term program sustainability is a problem. Given the renewed interest in controlling STH infections by 2020, and the commitment towards this effort by governments, donor agencies and pharmaceutical companies alike [55], this study assumes greater importance because of its potential to address some of the major shortfalls of the currently-practiced MDA strategy for STH control.

## Authors' contributions

RS, JM and GK conceived and designed the study and drafted the original protocol. AR helped in community sensitization, preparing the health education modules and supervised the field staff training. VR and VB helped revise the study protocol, and set up and maintain the geodatabases. SSRA supervised the laboratory assays. VV assisted with the study coordination and data management. RS was responsible for the clinical management of patients during the mass drug administration campaigns. VR and KG provided their expertise and guidance related to the clinical aspects of the study and in monitoring safety of the participants. RMA, NCG and SJB provided critical inputs on the study design, statistical analysis and model building. All authors read and approved the final manuscript.

## Funding

This study is funded by the Wellcome Trust/DBT India Alliance through an Early Career Fellowship to RS (Grant number: IA/E/12/1/500750). SJB is supported by a Wellcome Trust Senior Fellowship in Basic Biomedical Science (Grant number: 098045).

## Declaration of conflicting interests

RMA is a non-executive director of GlaxoSmithKline (GSK), one of the manufacturers of albendazole. The albendazole was purchased from the CMC pharmacy and GSK did not play any role in study design, data collection and analysis, decision to publish, or preparation of the manuscript.
